# The Big, the Dark, and the Biopsychosocial Shades of Harmony: Personality Traits and Harmony in Life

**DOI:** 10.3390/bs14100873

**Published:** 2024-09-27

**Authors:** Danilo Garcia

**Affiliations:** 1 Department of Social Sciences, University of Stavanger, 4021 Stavanger, Norway; danilo.garcia@icloud.com; 2 Lab for Biopsychosocial Personality Research (BPS-PR), International Network for Well-Being; 3 Promotion of Health and Innovation (PHI) Lab, International Network for Well-Being; 4 Department of Behavioral Sciences and Learning, Linköping University, 581 83 Linköping, Sweden; 5 Centre for Ethics, Law and Mental Health (CELAM), University of Gothenburg, 405 30 Gothenburg, Sweden; 6 Department of Psychology, University of Gothenburg, 405 30 Gothenburg, Sweden

**Keywords:** personality traits, subjective well-being, harmony in life, big five, biopsychosocial model, dark triad

## Abstract

Our current understanding of the relationship between personality traits and subjective well-being, or happiness, is limited to the conceptualization of subjective well-being as being life satisfaction and a positive affective experience (i.e., the presence of positive emotions and the absence of negative ones), thus lacking the sense of acceptance, balance, adaptation, and self-transcendent unity (i.e., harmony in life) that is appreciated as part of the good life in many ancient and modern cultures. Moreover, most studies use the Big Five Model to understand which personality traits predict subjective well-being. Here, I examine the predictive power of personality on harmony in life using the Big Five Model, the Dark Triad, and Cloninger’s Biopsychosocial Model. The present study utilized past published data from three cross-sectional studies. In each separate sample, participants self-reported personality by answering the Big Five Inventory (N_1_ = 297), the Short Dark Triad (N_2_ = 1876), or the Temperament and Character Inventory (N_3_ = 436). All participants (N_Total_ = 3698) answered to the Harmony in Life Scale. The traits in the Biopsychosocial Model explained the highest variance in harmony in life (*R*^2^ = 0.435, F(7, 428) = 47.136, *p* < 0.001), followed by the Big Five (*R*^2^ = 0.341, F(5, 291) = 30.110, *p* < 0.001) and the Dark Triad (*R*^2^ = 0.096, F(3, 1872) = 66.055, *p* < 0.001). The key significant predictors were Self-Directedness, Self-Transcendence, and Harm Avoidance from the Biopsychosocial Model and Agreeableness, Conscientiousness, and Neuroticism from the Big Five. Narcissism was the only predictor from the Dark Triad, although this relationship was very small. The findings underscore the importance of a multidimensional approach for understanding subjective well-being and the inclusion of harmony in life as its third component. The Biopsychosocial Model’s inclusion of both temperament and character dimensions provided the most comprehensive understanding of harmony in life. While positive traits like Agreeableness, Self-Directedness, and Self-Transcendence enhance harmony, negative traits like Neuroticism and Harm Avoidance diminish it. Moreover, research only including “dark traits” might give the impression that an inflated sense of self-importance, a deep need for admiration, and a lack of empathy for others (i.e., Narcissism) is predictive of balance in life. However, this association was not only extremely low but can be interpreted as misguided since the results using the other models showed that helpful, empathetic, kind, and self-transcendent behavior predicted harmony. These results suggest that interventions aimed at enhancing well-being should consider a broad range of personality traits, especially those that are not present in the Big Five Model, thus advocating for a biopsychosocial approach to well-being interventions.

## 1. Introduction

Subjective well-being, or happiness, is typically conceptualized as consisting of an affective component (i.e., positive and negative affect) and a cognitive component (i.e., life satisfaction) [1]. Due to its subjective nature, it is considered one of the best proxies for measuring, studying, and understanding human well-being and the impact of events, interventions, or public-policy decisions on quality of life [2]. Therefore, it is interesting to investigate what predicts subjective well-being. In this context, the relationship between personality and subjective well-being is a well-established area of research in psychology [3,4,5]. Traditionally, the traits in the Big Five Model of personality—Openness, Conscientiousness, Extraversion, Agreeableness, and Neuroticism [6]—have been central to this scientific discourse. These traits have been consistently linked to both components of subjective well-being. For instance, high levels of Extraversion and low levels of Neuroticism are strong predictors of frequently experiencing positive affect, infrequently experiencing negative affect, and feeling satisfied with life [3,4,5,7,8,9,10,11,12,13,14].

However, there are two concerns regarding our current understanding of the relationship between personality and subjective well-being. The first is that the original conceptualization of subjective well-being lacks a component of balance and congruence in one’s life, namely, harmony in life (i.e., a social or behavioral component). Indeed, when people in different cultures are asked about their conceptualizations of happiness, harmony in life seems to be the major factor people come to think about, even before life satisfaction or the presence of positive emotions and the absence of negative ones [15,16,17,18,19,20,21]. The second concern has its basis in the fact that research has found that long-lived and healthy individuals in different cultures are those who tend to experience positive emotions, are emotionally stable, conscientious, kind and empathetic, responsible, resourceful, self-acceptant, helpful, socially tolerant, and spiritual acceptant [22,23,24,25,26]. While some of the Big Five traits account for some of these individual differences, the Big Five Model has been criticized for mainly consisting of temperament traits or automatic emotional reactions [27,28,29,30,31]. Although temperament can be powerful and useful to describe and differentiate people, it does not account for what people make of themselves intentionally (i.e., goals and values or character). Indeed, character is the dimension of personality that helps people to regulate the emotional reactions derived from their temperament [32]. In other words, to understand why people behave the way they do we need to address differences within the individual’s unconscious (i.e., temperament) and conscious (i.e., character) levels of awareness [27,28,32,33,34,35,36,37,38,39,40,41,42]. Indeed, character traits, such as self-acceptance, adaptation, flexibility, resourcefulness, and spirituality, are probably how people achieve balance within the self and harmony in life.

### 1.1. Harmony in Life as a Third Component of Subjective Well-Being

Unlike the other subjective well-being components, which seem to emphasize individualistic pursuits like personal achievement or emotional states, harmony in life encompasses a broader, more integrative view of well-being [43,44]. It involves not only achieving personal goals and experiencing positive emotions but also maintaining psychological balance and flexibility. This balance is vital for navigating life’s complexities, allowing individuals to align the five planes of life (i.e., sexual, material, emotional, intellectual, spiritual) in a cohesive and fulfilling manner [45,46,47,48]. According to the theoretical framework proposed by Garcia and colleagues [45,49], harmony reflects a deeper level of well-being that transcends the mere presence of positive affect or satisfaction with specific life domains. More specifically, harmony in life is defined as a state of balance and congruence across the five planes of life, thus encompassing both internal psychological states and external social environments [45,49]. Harmony in life is particularly significant for understanding well-being within diverse cultural contexts, where values such as relational connectedness and inner peace are often prioritized. For instance, in many Eastern cultures, harmony is a central value, often taking precedence over individualistic pursuits like personal achievement [15,17].

Several empirical studies provide robust evidence supporting the significance of harmony in life as a distinct and critical component of subjective well-being. For instance, harmony in life has been shown to be associated with high levels of psychological well-being but also as the main component of a general subjective well-being factor [49,50]. Hence, harmony in life offers a comprehensive framework for understanding and enhancing subjective well-being. It emphasizes the importance of balance, coherence, and integration, providing valuable insights for both theoretical models and practical applications in the science of well-being.

### 1.2. Two Alternative Models of Personality

Broadly speaking, personality has to do with people’s motivation, way of learning, and mental adaptation, which is probably why it is the major single predictor of subjective well-being [32]. In other words, our personality contributes to both inter- and intrapersonal relations in the five different planes of life [32,48,51,52]. Therefore, even if the Big Five provides a useful framework for understanding basic personality traits, it may not fully capture the complexity of how personality influences well-being. Fortunately, recent research suggests that alternative models of personality, such as the Dark Triad [53,54] and Cloninger’s Biopsychosocial Model [32,55], provide additional insights into the complex relationships between personality and well-being.

The Dark Triad—Machiavellianism, Narcissism, and Psychopathy—provides a contrasting perspective to the Big Five Model because it focuses on socially aversive traits [53]. Research using the Dark Triad has shown that these traits have nuanced and sometimes contradictory effects on subjective well-being. For instance, while Machiavellianism and Psychopathy are generally associated with lower levels of subjective well-being, probably due to their negative social and interpersonal impacts, Narcissism has been found to correlate positively with life satisfaction and positive affect [56,57]. This positive correlation is often attributed to the self-enhancing and confidence-boosting nature of narcissism, which can lead to perceived social success and inflated self-esteem [58]. Hence, the inclusion of the Dark Triad traits in the study of personality and well-being might be crucial for a more comprehensive understanding of how different personality dimensions influence an individual’s attitudes towards life.

The Biopsychosocial Model offers a more holistic perspective on personality by decomposing it and distinguishing between temperament and character dimensions [59,60]. This model, proposed by Cloninger and colleagues [55], emphasizes the interplay between biological, psychological, and social factors in shaping personality and behavior. Temperament refers to the automatic emotional responses that are largely biologically driven, while character involves self-concept and values shaped by learning and social interactions [22,32]. The four temperament traits are: Novelty Seeking, Harm Avoidance, Reward Dependence, and Persistence. Three character traits, Self-Directedness, Cooperativeness, and Self-Transcendence, are crucial for achieving harmony in life, as they facilitate the alignment of actions with personal goals and values, fostering a coherent and fulfilling life experience [61,62,63,64,65]. Indeed, studies using the Temperament and Character Inventory (TCI) have demonstrated that these character traits are strongly predictive of subjective well-being, including harmony in life.

See Table 1 for an overview of the theories, definitions, measures, and methodology behind the three personality models in the present set of studies.

### 1.3. The Present Study

In this study, I aimed to investigate the relationship between personality and harmony in life, an often-overlooked subjective well-being component using three distinct models (i.e., the Big Five, the Dark Triad, and Cloninger’s Biopsychosocial Model) to obtain a deeper understanding of the diverse pathways through which personality influences well-being. More specifically, the research question is: what is the predictive power of personality on harmony in life using the Big Five Model, the Dark Triad, and Cloninger’s Biopsychosocial Model?

## 2. Method

### 2.1. Participants and Procedure

The study involved 3698 participants recruited through Amazon’s Mechanical Turk (MTurk), a platform known for its efficiency in providing diverse and high-quality data [68,69,70]. Part of the dataset has been published in previous studies but has never been analyzed regarding the association between personality and harmony in life [49,58,71]. Participants were U.S. residents with English as their native language. The samples consisted of 297 participants (*N*_1_) in the Big Five model cohort, 1876 (*N*_2_) in the Dark Triad cohort, and 436 (*N*_3_) in the Biopsychosocial Model cohort. All 3698 participants self-reported harmony in life. Demographic data including age, gender, cultural background, and socioeconomic status was collected to control for potential confounding variables.

Participants were compensated USD 1.00 for completing the survey. To ensure data quality, the survey included three attention-check questions (e.g., “This is a control question, please answer “neither agree nor disagree”). Additionally, the time taken to complete the survey was electronically monitored. Data from participants who failed any of the attention-check questions or completed the survey in under 10 min were excluded from the analysis.

### 2.2. Measures

#### 2.2.1. Personality

The Big Five Inventory [72] was used to measure the Big Five personality traits: Openness (e.g., “I have active imagination”), Conscientiousness (e.g., “I persevere until the task is finish”), Extraversion (e.g., “I am talkative”), Agreeableness (“I am helpful and unselfish with others”), and Neuroticism (e.g., “I get nervous easily”). It includes 44 items rated on a 5-point Likert scale (1 = *Strongly Disagree*, 5 = *Strongly Agree*). Internal inconsistency in the present study, measured as *Cronbach’s alpha*, was 0.76 for Openness, 0.79 for Conscientiousness, 0.86 for Extraversion, 0.78 for Agreeableness, and 0.86 for Neuroticism.

The Short Dark Triad [73,74,75] was used to assess the Dark Triad traits—Machiavellianism (e.g., “Most people can be manipulated”), Narcissism (e.g., “I insist on getting the respect I deserve”), and Psychopathy (e.g., “It’s true that I can be mean to others”)—using 27 items, rated on a 5-point Likert scale (1 = *Strongly Disagree*, 5 = *Strongly Agree*). Internal inconsistency in the present study, measured as *Cronbach’s alpha*, was 0.75 for Machiavellianism, 0.73 for Narcissism, and 0.72 for Psychopathy.

The Temperament and Character Inventory (TCI) 60-item version [61] was used to measure Cloninger’s four temperament dimensions—Novelty Seeking (e.g., “I often try new things just for fun or thrills, even if most people think it is a waste of time”), Harm Avoidance (e.g., “I often feel tense and worried in unfamiliar situations, even when others feel there is little to worry about”), Reward Dependence (e.g., “I like to discuss my experiences and feelings openly with friends instead of keeping them to myself”), and Persistence (e.g., “I often push myself to the point of exhaustion or try to do more than I really can”)—and three character dimensions—Self-Directedness (e.g., “In most situations, my natural responses are based on good habits that I have developed”), Cooperativeness (e.g., “I often consider another person’s feelings as much as my own”), and Self-Transcendence (e.g., “I sometimes feel so connected to nature that everything seems to be part of one living organism”). The items are rated on a 5-point Likert scale (1 = *Definitely False*, 5 = *Definitely True*). Internal inconsistency in the present study, measured as *Cronbach’s alpha*, was 0.65 for Novelty Seeking, 0.86 for Harm Avoidance, 0.65 for Reward Dependence, 0.82 for Persistence, 0.87 for Self-Directedness, 0.76 for Cooperativeness, and 0.71 for Self-Transcendence.

#### 2.2.2. Harmony in Life

The Harmony in Life Scale [44] was used to assess perceived harmony in life. It consists of 5 items (e.g., “My lifestyle is well balanced”, “I feel that my life is in harmony”) rated on a 7-point Likert scale (1 = *Strongly Disagree*, 7 = *Strongly Agree*). Internal inconsistency in the present study, measured as *Cronbach’s alpha*, in the different samples was *N*_1_ = 0.90, *N*_2_ = 0.89, and *N*_3_ = 0.94.

## 3. Results

### 3.1. Correlation Analyses

The correlation analyses examined the relationships between harmony in life across the three different samples: Big Five, Dark Triad, and the Biopsychosocial Model.

Regarding the Big Five (see Table 2), the analysis showed that, as expected, Conscientiousness, Extraversion, and Agreeableness are moderately positively correlated with harmony in life, while Neuroticism is strongly negatively correlated. However, Openness did not have a significant relationship with harmony in life. Regarding the Dark Triad traits (see Table 3), Narcissism showed a small positive correlation with harmony in life, while Machiavellianism and Psychopathy showed small negative correlations. Nevertheless, all Dark Triad correlations were below 0.20, which is the recommended minimum effect size representing a “practically” significant effect for social science data [76]. Finally, regarding the Biopsychosocial Model’s temperament traits (see Table 4), Novelty Seeking did not show a significant correlation with harmony in life, Harm Avoidance exhibited a strong negative correlation, Reward Dependence a small positive correlation, and Persistence a moderate positive correlation. For the Biopsychosocial Model’s character traits (see Table 4), Self-Directedness exhibited a strong positive correlation, Cooperativeness a small positive correlation, and Self-Transcendence a small positive correlation.

### 3.2. Regression Analyses

The analysis of the Big Five personality traits revealed that these traits collectively accounted for 34.1% of the variance in harmony. The significant predictors were Agreeableness and Conscientiousness, which were both positively correlated with harmony, and Neuroticism, which was negatively associated with harmony. Extraversion and Openness did not significantly predict harmony in life (see Table 4). The Dark Triad analysis showed that these traits explained 9.6% of the variance in harmony in life. Narcissism predicted high harmony in life scores, while both Machiavellianism and Psychopathy predicted low scores (see Table 4). Lastly, the analysis using the Biopsychosocial Model of personality showed that these traits accounted for 43.5% of the variance in harmony in life. The significant positive predictors were Self-Directedness and Self-Transcendence, while Harm Avoidance predicted low harmony in life scores. The other personality traits in the Biopsychosocial Model, Novelty Seeking, Reward Dependence, Persistence, and Cooperativeness, did not show significant predictive value for harmony in life. See Table 5 for the details.

### 3.3. Comparison of Models Based on R^2^ Values

Using the Fisher r-to-z transformation, the effect of the traits in the different models was statistically compared. The effect of the Big Five traits on harmony (*R*^2^ = 0.341, *z* = 0.6684) was significantly larger (*z* = 4.13, *p* < 0.001) than the effect of the dark traits (*R*^2^ = 0.096, *z* = 0.3204). Likewise, the effect of the traits in the Biopsychosocial Model on harmony (*R*^2^ = 0.435, *z* = 0.7920) was larger (*z* = 6.93, *p* < 0.001) than the effect of the dark traits (*R*^2^ = 0.096, *z* = 0.3204). In contrast, the effect of the Big Five traits on harmony (*R*^2^ = 0.341, *z* = 0.6684) was smaller, although not significantly (*z* = −1.47, *p* = 0.07), than the effect of the Biopsychosocial Model traits (*R*^2^ = 0.435, *z* = 0.7920).

## 4. Discussion

The aim of the present study was to investigate the relationship between subjective well-being and personality by focusing on harmony in life and three different personality models: the Big Five, the Dark Triad, and the Biopsychosocial Model. The results not only corroborate established associations within the literature but also offer new insights into how specific traits contribute to or detract from individuals’ sense of balance and coherence in their lives.

The Big Five personality traits accounted for a significant proportion of the variance in harmony in life (34.1%). Among these traits, Agreeableness and Conscientiousness were strong positive predictors, which is consistent with prior research indicating that these traits are linked to prosocial behavior, reliability, and effective life management—all of which contribute to a balanced and harmonious life [8,77]. Also in this line, the negative association between Neuroticism and harmony underscores the disruptive impact of emotional instability and stress reactivity on one’s ability to maintain a coherent and fulfilling life. This finding aligns with extensive evidence suggesting that high levels of Neuroticism are detrimental to overall well-being [9]. Moreover, the lack of significant effects for Extraversion and Openness suggests that while these traits influence other aspects of subjective well-being, they are less central to the concept of harmony in life.

The Dark Triad traits had complex interactions with harmony in life. The positive correlations between Narcissism and harmony suggests that traits associated with high self-esteem and confidence can contribute to a subjective sense of harmony, possibly through successful social manipulation or self-promotion [56,78]. However, the negative associations found for Machiavellianism and Psychopathy indicate that these traits, characterized by manipulativeness and a lack of empathy, are generally detrimental to achieving a harmonious life. These findings are consistent with the notion that dark traits can disrupt interpersonal relationships and create conflict, which undermines overall harmony and well-being [79]. Nevertheless, all Dark Triad correlations were below 0.20, which is the recommended minimum effect size representing a “practically” significant effect for social science data [76]. This is remarkable in many ways, since it was expected that these traits will significantly decrease harmony in life due to their antisocial nature.

Cloninger’s Biopsychosocial Model provided the most comprehensive explanation for harmony in life, explaining 43.5% of the variance. This model’s strength lies in its integration of both temperament and character dimensions, allowing for a broader exploration of personality [33,34,80]. Self-Directedness and Self-Transcendence emerged as the strongest positive predictors, reinforcing the importance of these character traits in achieving life harmony. Self-Directedness involves autonomy, self-acceptance, responsibility, resourcefulness, and goal-directedness, which are crucial for navigating life challenges and achieving a balanced lifestyle [32]. Self-Transcendence, reflecting spirituality and a sense of connectedness to a larger existence, supports the attainment of inner peace and coherence [51,52,81,82,83]. The negative association with Harm Avoidance suggests that excessive caution and fear can hinder the pursuit of meaningful life experiences, thus reducing harmony. These findings align with the biopsychosocial perspective that both innate and learned aspects of personality are vital for well-being [49,61].

Hence, as expected, personality had a large effect on the social component of subjective well-being. Nevertheless, recent research clearly shows that subjective well-being is a multidimensional construct [49,50,84,85,86,87,88,89] that is best understood as a latent factor that is influenced and influences the cognitive (i.e., life satisfaction), the affective (i.e., positive and negative emotions), and the social (i.e., harmony in life) component. As the matter of fact, personality is related to this latent factor and then to its different components [8]. If so, merely analyzing the relationship between personality and harmony in life might only be telling half of the story. Which explains why the dark traits where so weakly related to harmony in life. To fully understand this relationship is important to account for the other components of subjective well-being.

### 4.1. Limitations

While this study provides comprehensive insights into the relationships between personality traits and harmony in life, several limitations must be acknowledged. The study’s cross-sectional nature limits the ability to infer causality. The findings represent correlations between personality traits and harmony at a single point in time and in different samples, thus making it challenging to determine the direction of these relationships. To further understand the predictive power of each model, additional statistical techniques, such as hierarchical regression or comparative fit indices, could have been applied if all participants had answered to instruments measuring all three personality models. Likewise, longitudinal studies are needed to establish causal pathways and examine how changes in personality traits may impact harmony in life over time [90].

Moreover, the present study relied on self-reported data for assessing personality traits and harmony in life, which introduces the potential for response biases, such as social desirability or self-enhancement biases. These biases could lead to inflated or deflated reporting of traits and well-being measures, potentially skewing the results. Nevertheless, the measure used for the Biopsychosocial Model, the Temperament and Character Inventory, includes scales for detecting response bias (i.e., performance scales). These scales have been shown to be extremely accurate for detecting deceiving, random, and other types of problematic responses.

Another possible caveat is that the sample was recruited via MTurk, which may not be fully representative of the general population. While MTurk provides access to a diverse participant pool, it can also over-represent certain demographics, such as younger, more educated, and technologically adept individuals. This limitation may affect the generalizability of the findings to broader and more varied populations [69]. Nevertheless, the population in the present study was diverse [49,58,71]. Last but not the least, although the study incorporated three comprehensive models, there are other relevant personality frameworks and dimensions that where not examined, such as the HEXACO model. The HEXACO model integrates the trait of Honesty-Humility besides the traits included in the Big Five [91,92,93,94,95,96,97,98]. Indeed, humble individuals are likely to maintain balanced and respectful interpersonal relationships, which is crucial for harmony in life. Moreover, Honesty-Humility’s emphasis on low self-focus and an appreciation for others can enhance emotional regulation and congruence between one’s internal values and external behaviors. Humble individuals are less likely to experience conflicts stemming from ego-driven decisions or entitlement, leading to a more balanced and coherent sense of self and life satisfaction [91]. Overall, Honesty-Humility fosters an environment where personal goals and values align with respectful and fair treatment of others, supporting a coherent and fulfilling life experience that aligns well with the concept of harmony. Although this description of Honesty-Humility mirrors certain sub-traits within the trait of Self-Transcendence in the Biopsychosocial Model [60,83], including the HEXACO model could provide additional insights and a more nuanced understanding of the relationships between personality and subjective well-being.

### 4.2. Conclusions and Last Remarks

This study underscores the importance of a multidimensional approach to understanding subjective well-being and the inclusion of harmony in life as a third component. In sum, positive traits like Agreeableness, Self-Directedness, and Self-Transcendence enhance harmony, while negative traits like Neuroticism and Harm Avoidance diminish it. The Biopsychosocial Model’s inclusion of both temperament and character dimensions provided the most comprehensive understanding of harmony in life. Moreover, only including “dark traits” might give the impression that an inflated sense of self-importance, a deep need for admiration, and a lack of empathy for others (i.e., Narcissism) is predictive of balance in life. However, this association was not only extremely low but can be interpreted as misguided since the results using the other models showed that helpful, empathetic, kind, and self-transcendent behavior was predictive of harmony; all of which are negatively associated with Narcissism.

These results suggest that interventions aimed at enhancing well-being should consider a broad range of personality traits, especially those that are not present in the Big Five Model, thus advocating for a biopsychosocial approach to well-being interventions. For instance, increasing Self-Directedness, Cooperativeness, and Self-Transcendence and reducing Harm Avoidance might be particularly effective in promoting a harmonious life. Such interventions have indeed been successful in reducing depression, anxiety, and other types of mental illness while increasing life satisfaction, positive emotions, and harmony in life [61,99,100,101,102,103,104]. These interventions promote psychological flexibility, emotional regulation, and a sense of coherence by including mindfulness practices, cognitive behavioral strategies, and programs aimed at fostering personal growth, self-acceptance, and spiritual acceptance (see www.anthropedia.org). By targeting the integration of diverse life aspects, such interventions lead to more sustainable improvements in well-being, beyond what is achieved by focusing solely on positive emotions or specific life domains.

## Figures and Tables

**Table 1:** Overview of the theories, definitions, measures, and methodology behind the three personality models in the present set of studies.

Theory	Definition of Personality	Hypotheses	Model	Dimensions	Traits	Measurement	Example of Questions	Method
**NOT APPLICABLE**	Personality consists of recurring characteristics in the form of emotions, thoughts, and behaviors that distinguish one person from others.	Relevant and prominent characteristics of the individual encoded in language [66].	Big Five	Temperament (+ Character to some extent, especially prosocial traits)	**O**penness **C**onscientiousness **E**xtraversion **A**greeableness **N**euroticism	- The Revised NEO Personality Inventory Revised (NEO-PI-R) - Big Five Inventory (BFI) - Big Five Inventory 2 (BFI-2) - Ten Item Personality Measure - Etcetera.	**O**: I have active imagination. **C**: I persevere until the task is finish. **E**: I am talkative. **A**: I am helpful and unselfish with others. **N**: I get nervous easily.	Factor Analysis
**EVOLUTION**	Personality is an adaptive system of traits that enhance an individual’s ability to navigate social hierarchies, exploit opportunities, and manage interpersonal relationships in ways that maximize personal gains and reproductive success.	The propensity for manipulation, risk-taking, and self-aggrandizement has advantageous in certain environmental contexts [53].	Dark Triad	Temperament (+Character to some extent, especially the lack of prosocial traits)	**M**achiavellianism **N**arcissism **P**sychopathy	- The Short Dark Triad (SD3) - The Dark Trad Dirty Dozen (DTTD) - Mach-IV - Narcissistic Personality Inventory (NPI) - Etcetera.	**M**: Most people can be manipulated. **N**: I insist on getting the respect I deserve. **P**: It’s true that I can be mean to others.	
	Personality is the dynamic organization of biopsychosocial systems within an individual through which the individual uniquely shapes and adapts to an ever-changing internal and external environment.	- A person’s thoughts, feelings, and behavior are influenced roughly equally by the person’s body, mind, and psyche. - It is possible to quantify both the processes within individuals and the external processes that affect their ways of adapting to life experiences. - Changes in an individual’s thoughts, feelings, and behavior are dynamic expressions of complex adaptive systems that evolves gently from moment to moment and can be characterized not only by their means but also by their range. - It is possible to identify specific clusters of genes and brain networks activated by the psychobiological processes underlying person–situation interactions and to show that these non-linear dynamic interactions are strongly correlated with individual differences in personality dimensions. - Etcetera [67].	Cloninger’s Biopsychosocial Model	Temperament	**N**ovelty **S**eeking **H**arm **A**voidance **R**eward **D**ependence **P**ersistence	- The Tridimensional Personality Questionnaire (TPQ) - The Temperament and Character Inventory (TCI)	**NS:** I often try new things just for fun or thrills, even if most people think it is a waste of time. **HA:** I often feel tense and worried in unfamiliar situations, even when others feel there is little to worry about. **RD:** I like to discuss my experiences and feelings openly with friends instead of keeping them to myself. **PS:** I often push myself to the point of exhaustion or try to do more than I really can.	- Molecular Genetics - Confirmatory Factor Analysis - Non-Linear Factor Analysis - Latent Profile Analysis - Latent Class Analysis
				Character	**S**elf-**D**irectedness **C**ooperativeness **S**elf-**T**ranscendence	- The Temperament and Character Inventory (TCI)	**SD**: In most situations, my natural responses are based on good habits that I have developed. **CO**: I often consider another person’s feelings as much as my own. **ST**: I sometimes feel so connected to nature that everything seems to be part of one living organism.	

**Table 2:** Correlations between the Big Five traits and harmony in life (*N*_1_ = 296).

Big Five Traits	1	2	3	4	5	6
Openness (1)						
Conscientiousness (2)	0.141 *					
Extraversion (3)	0.214 **	0.164 **				
Agreeableness (4)	0.129 *	0.436 **	0.207 **			
Neuroticism (5)	−0.060	−0.450 **	−0.311 **	-0.426 **		
Harmony in Life (6)	0.041	0.391 **	0.231 **	0.390 **	−0.541 **	

Note: * *p* < 0.01; ** *p* < 0.001. Blue Cells: Significant correlations between Big Five traits and harmony in life.

**Table 3:** Correlations between Dark Triad traits and harmony in life (*N*_2_ = 1875).

Dark Traits	1	2	3	4
Machiavellianism (1)				
Narcissism (2)	0.328 **			
Psychopathy (3)	0.533 **	0.394 **		
Harmony in Life (4)	−0.134 **	0.164 **	−0.156 **	

Note: ** *p* < 0.001. Black Cells: Significant correlations between dark traits and harmony in life.

**Table 4:** Correlations between temperament and character traits (i.e., Cloninger’s Biopsychosocial Model) and harmony in life (*N*_3_ = 436).

TCI Traits	1	2	3	4	5	5	7	8
Novelty Seeking (1)								
Harm Avoidance (2)	0.059							
Reward Dependence (3)	−0.004	−0.169 **						
Persistence (4)	−0.153 **	−0.557 **	0.213 **					
Self−Directedness (5)	−0.335 **	−0.739 **	0.209 **	0.585 **				
Cooperativeness (6)	−0.317 **	−0.195 **	0.450 **	0.201 **	0.293 **			
Self−Transcendence (7)	0.183 **	−0.050	0.216 **	0.152 **	0.011	0.145 **		
Harmony in Life (8)	−0.069	−0.555 **	0.234 **	0.443 **	0.590 **	0.221 **	0.236 **	

Note: ** *p* < 0.001.TCI = Temperament and Character Inventory. Purple Cells: Significant correlations between temperament and character traits and harmony in life.

**Table 5:** Regression analyses, one for each personality model and sample, using personality traits as the predictors and harmony in life as the outcome variable.

Model	Trait	*r* (*p*)	*Adj R* ^2^	*Unst. B*	*Unst. SE*	*Stand. β*	*F*	*t*	*p*
**BIG FIVE**	Openness	0.04 (0.239)	0.341	−0.01	0.01	−0.04	30.11	−0.70	0.482
	Conscientiousness	0.39 (<0.001)		0.03	0.01	0.14		2.57	0.011
	Extraversion	0.23 (<0.001)		0.01	0.01	0.06		1.16	0.247
	Agreeableness	0.39 (<0.001)		0.04	0.01	0.15		2.71	0.01
	Neuroticism	−0.54 (<0.001)		−0.08	0.01	−0.40		−6.85	<0.001
**DARK TRIAD**	Machiavellianism	−0.13 (<0.001)	0.096	−0.26	0.05	−0.13	66.05	−4.90	<0.001
	Narcissism	0.16 (<0.001)		0.61	0.05	0.29		11.83	<0.001
	Psychopathy	−0.16 (<0.001)		−0.48	0.06	−0.20		−7.63	<0.001
**BIOPSYCHOSOCIAL**	Novelty Seeking	−0.07 (0.075)	0.435	0.14	0.10	0.06	47.14	1.37	0.170
	Harm Avoidance	−0.56 (<0.001)		−0.29	0.08	−0.20		−3.52	<0.001
	Reward Dependence	0.23 (<0.001)		0.11	0.08	0.05		1.29	0.198
	Persistence	0.44 (<0.001)		0.10	0.09	0.06		1.20	0.232
	Self−Directedness	0.59 (<0.001)		0.68	0.11	0.41		6.50	<0.001
	Cooperativeness	0.22 (<0.001)		0.04	0.10	0.02		0.40	0.690
	Self−Transcendence	0.24 (<0.001)		0.33	0.07	0.19		4.85	<0.001

## Data Availability

The datasets used and/or analyzed during the current study are available from the corresponding author on reasonable request.
